# Supplementary figures and images for: The Genetic Legacy of the Expansion of Turkic-Speaking Nomads across Eurasia
Source: PLoS Genet. 2015 Apr 21;11(4):e1005068. doi: 10.1371/journal.pgen.1005068 (PMC4405460; doi:10.1371/journal.pgen.1005068)

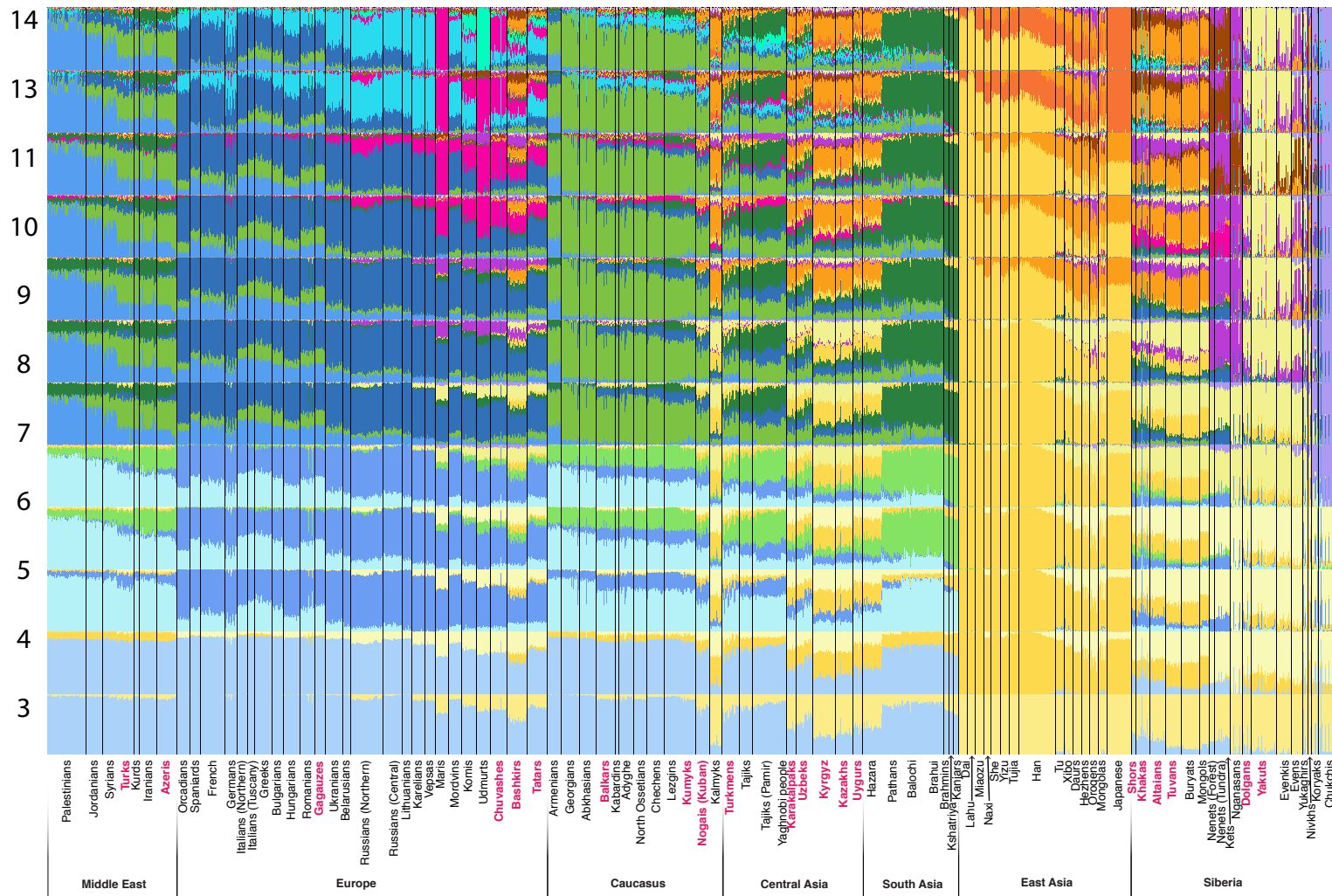

Supplement: S1 Fig — Each individual is represented by a vertical (100%) stacked column indicating the proportions of ancestry in K constructed ancestral populations. The models (K) shown here likely each converged to a global likelihood maximum as > 10% of runs (100 replicates in total for each K) with the highest log-likelihood (LL) converged to essentially to the same solution with a log-likelihood difference of > 1 LL units. We plotted the runs with the highest LL at each K. (PDF) [file pgen.1005068.s001.pdf]

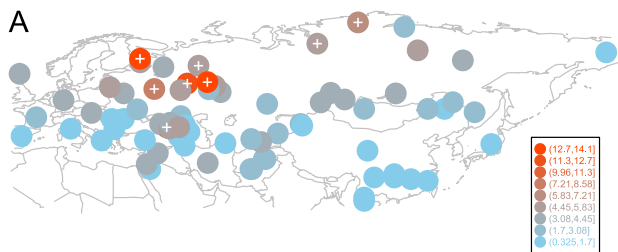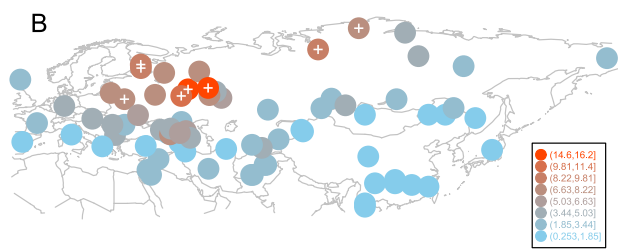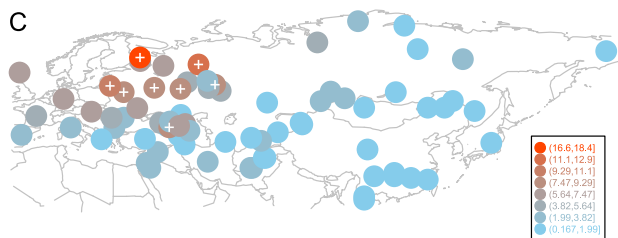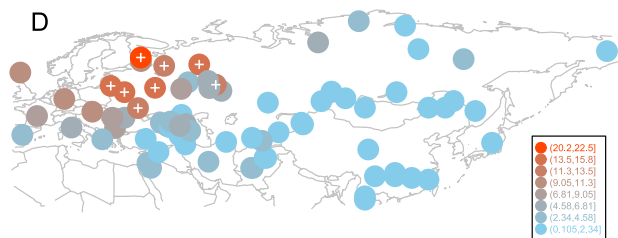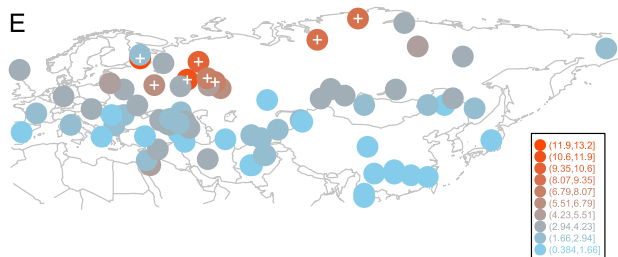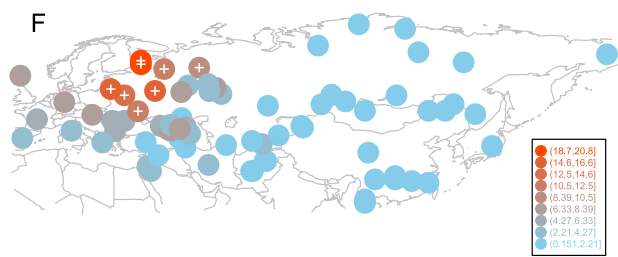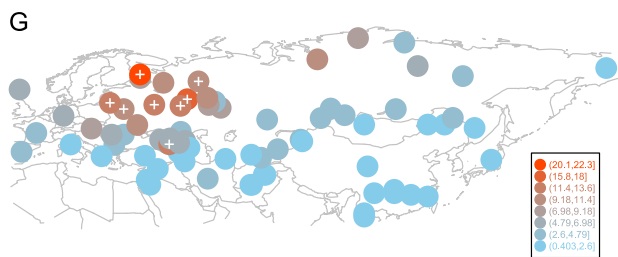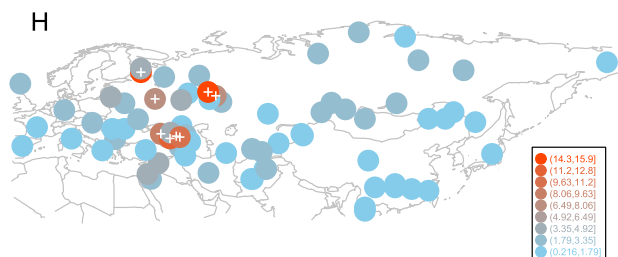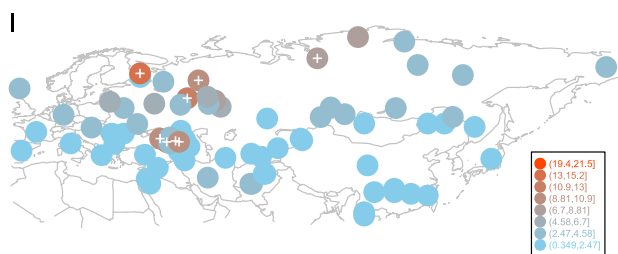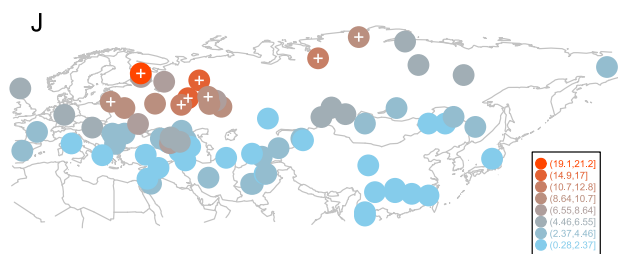

Supplement: S3 Fig — Circle position corresponds to population location. Circle color indicates the amount of excess IBD sharing (shown in the Legend) that this population shares with all 12 randomly selected geographic neighbors. Populations with IBD sharing exceeding the 0.90 quantile are shown with the “plus symbol”. Panels A–J show IBD sharing signals for different randomly selected combinations of geographic neighbors. All the results are based on IBD tracts of 1–2 cM. (PDF) [file pgen.1005068.s003.pdf]

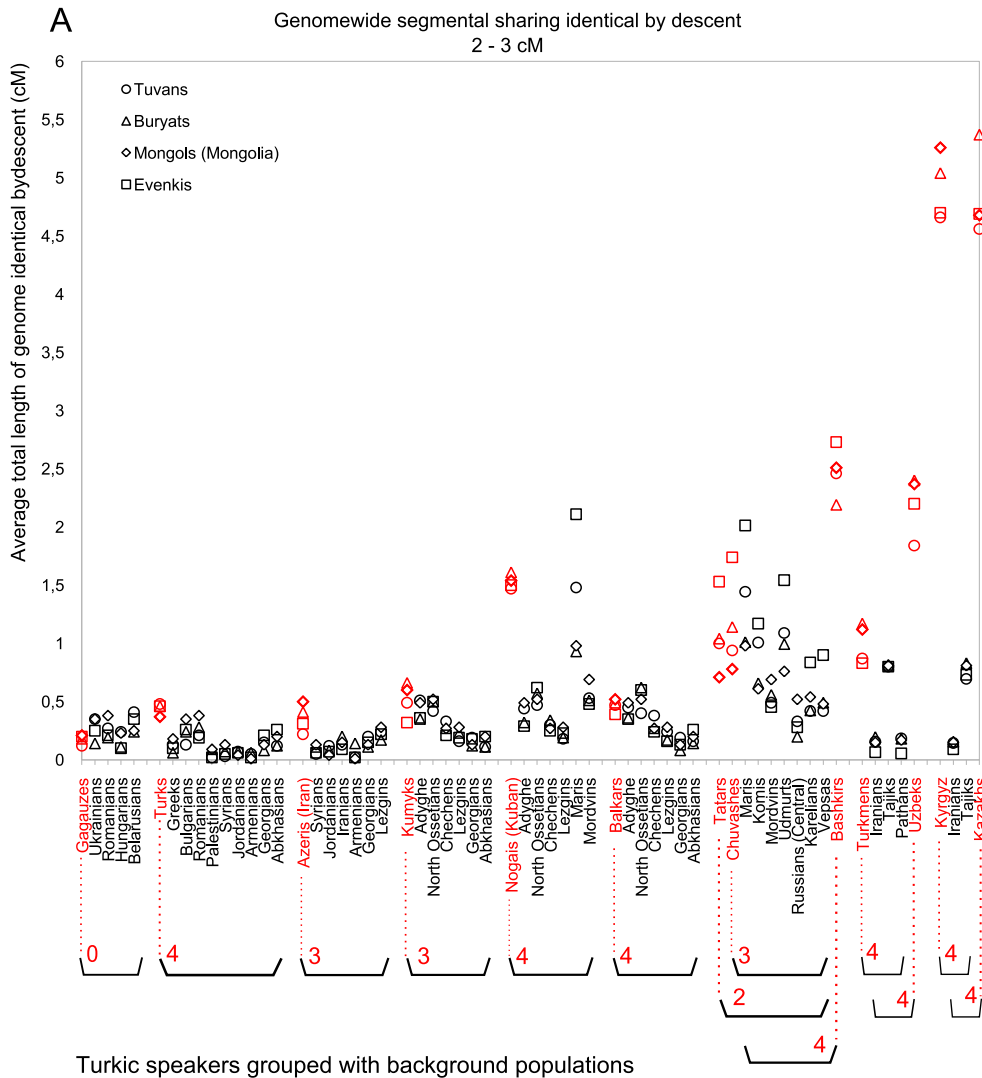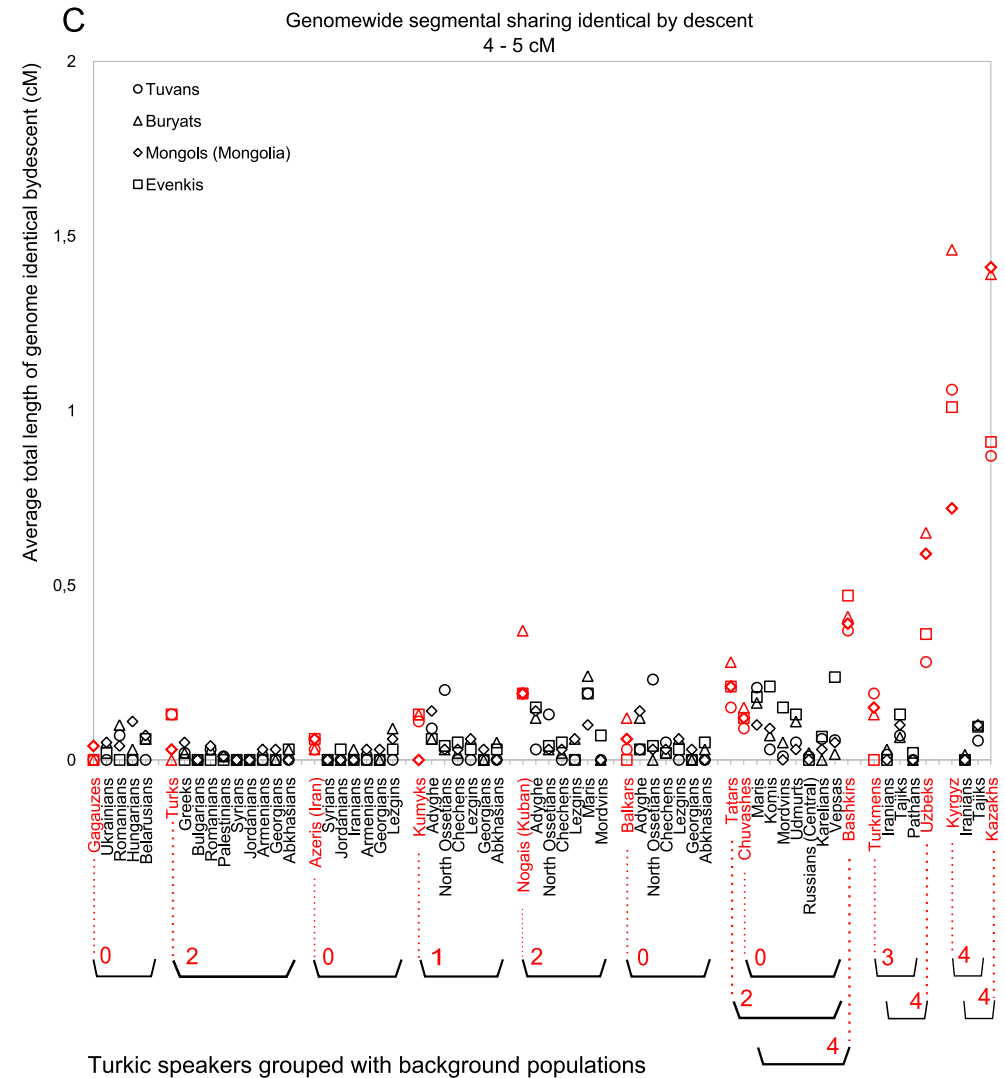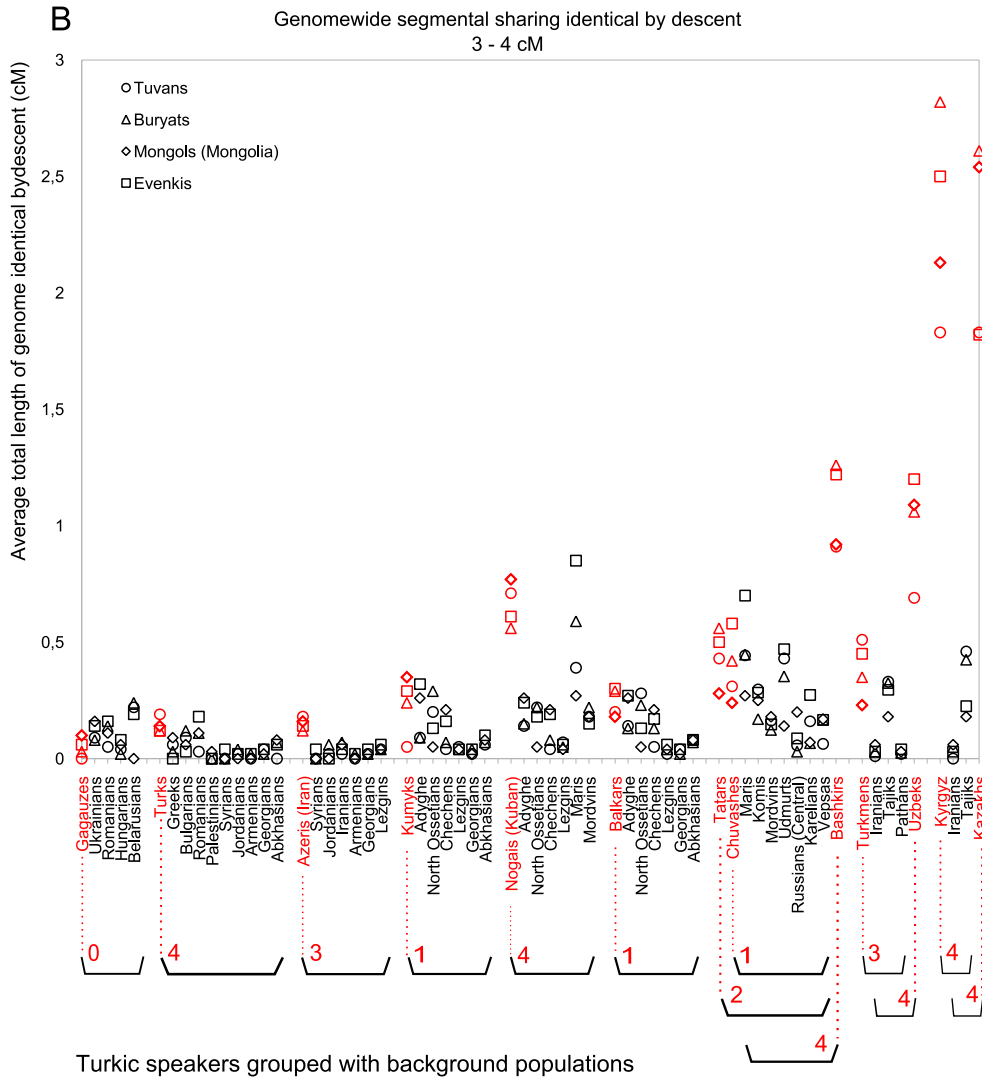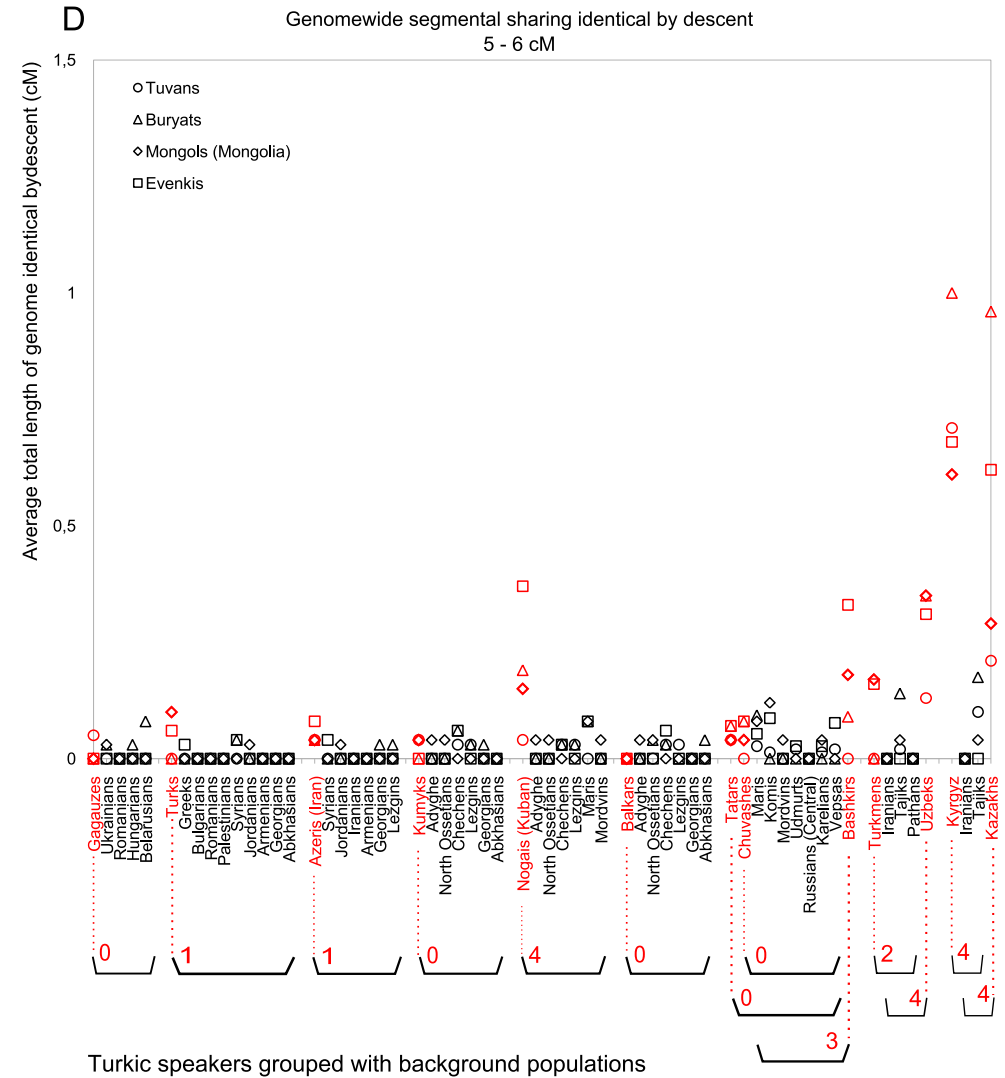

Supplement: S4 Fig — Panels A, B, C, and D show results for different segment size bins (2–3, 3–4, 4–5, 5–6 cM). For each population ordered along the x–axis, IBD sharing is computed with four populations (Tuvans, Buryats, Mongols (Mongolia), and Evenkis) from the SSM area. Each Turkic-speaking population (shown in red) is grouped with its respective geographic neighbors using parentheses. The grouped geographic neighbors were pooled and used to perform a permutation test as described in the M&M section. The red number under the Turkic population name shows how many SSM populations demonstrate a statistically significant excess of IBD sharing with a given Turkic population. Overlapping parentheses show Turkic-speaking populations with shared geographic neighbors. (PDF) [file pgen.1005068.s004.pdf]

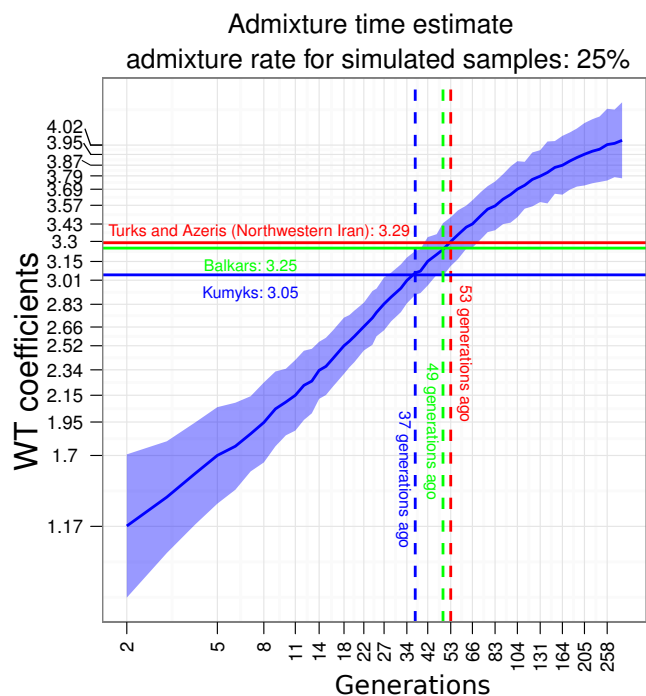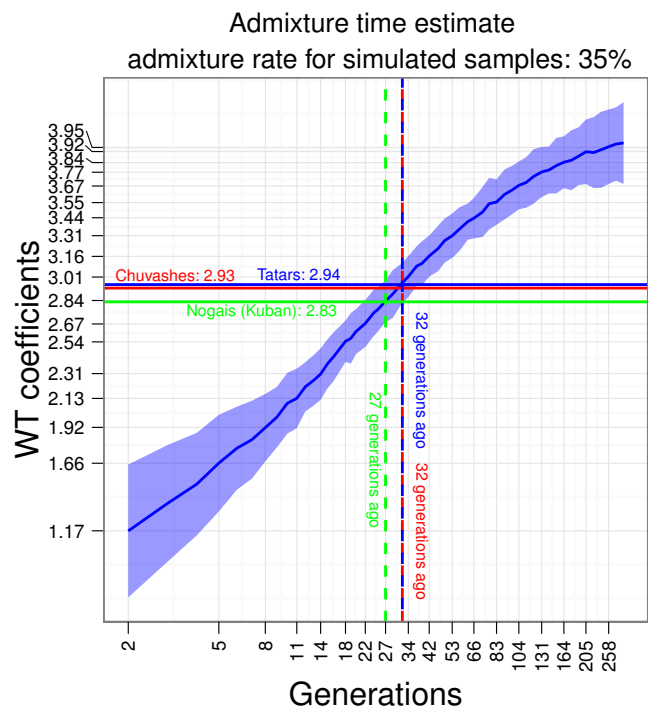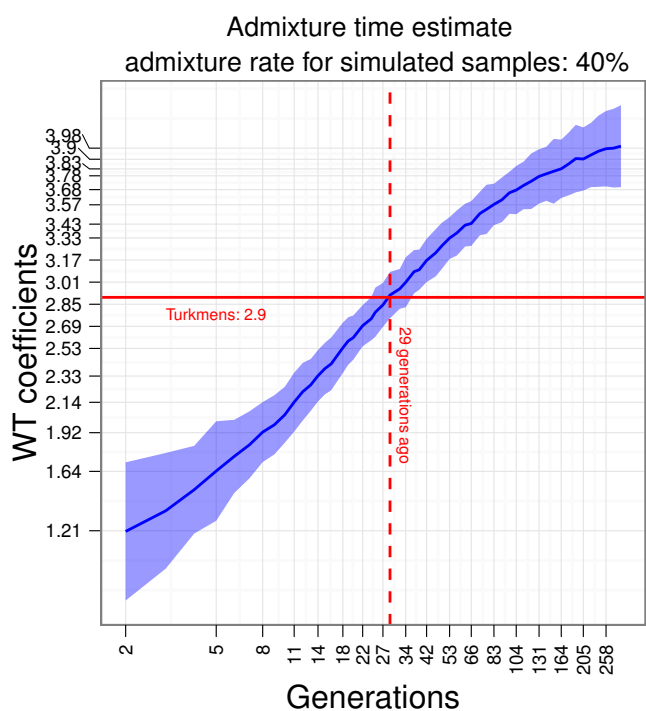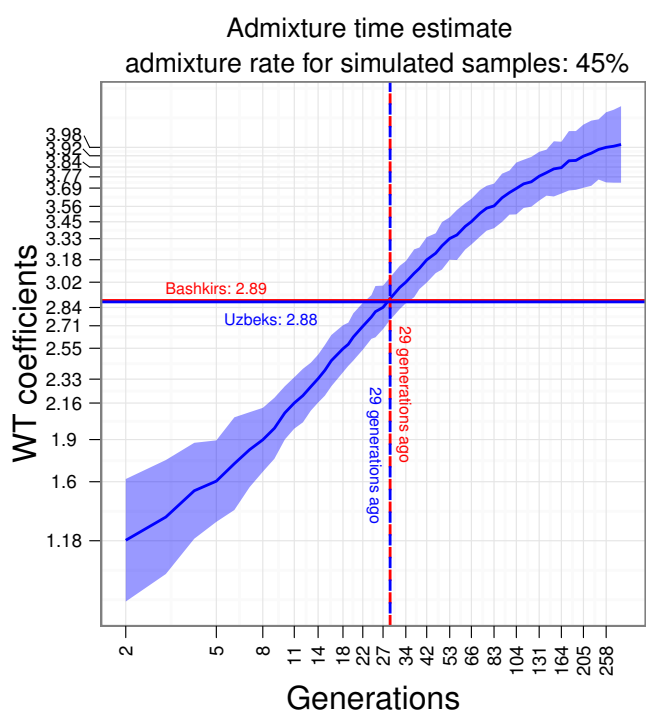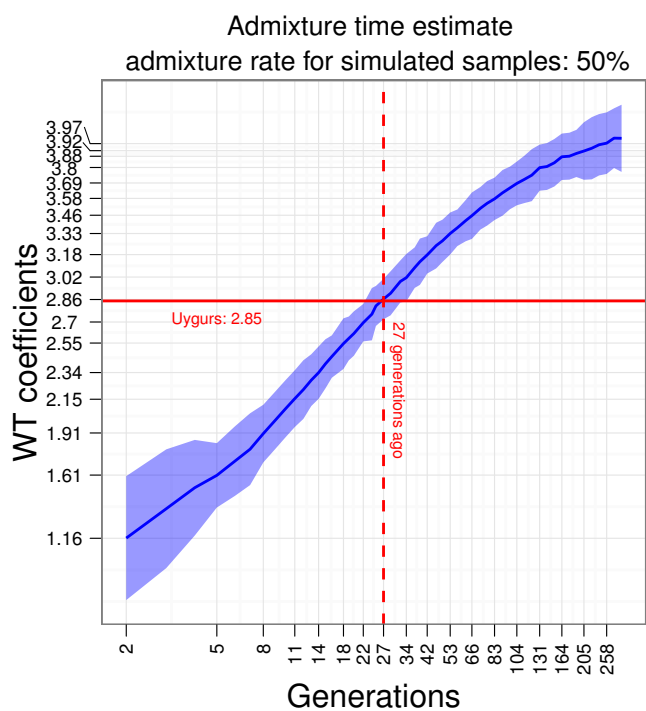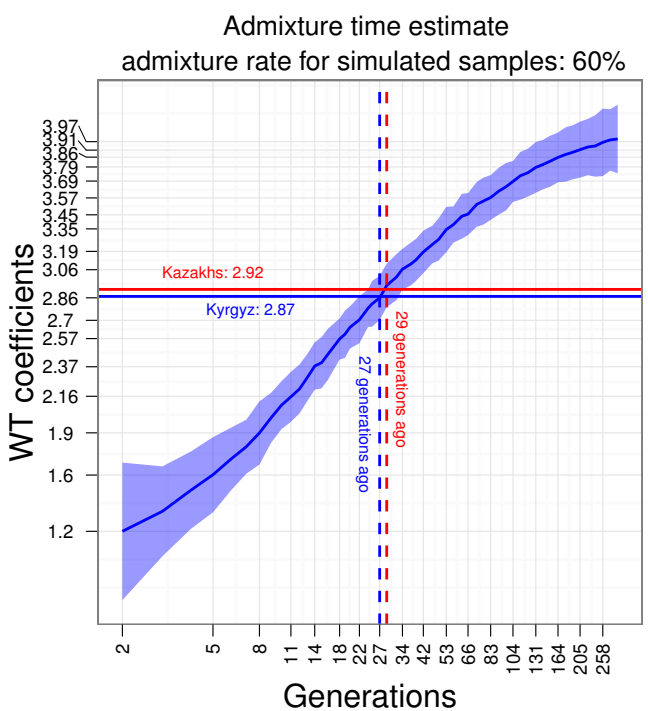

Supplement: S5 Fig — The blue curve shows the relationship between the WT coefficient and time since admixture in a growing population. Each curve summarizes the outcome from 100 forward simulations. Thus, the bold curve shows the average WT coefficient over 100 simulations and the blue shaded area shows the 95% confidence interval. Horizontal lines in red, green, and blue show point estimates of the WT coefficient for different Turkic-speaking populations. The intersection point between the horizontal line and the blue curve gives the admixture time estimate, shown with dashed vertical lines. (PDF) [file pgen.1005068.s005.pdf]

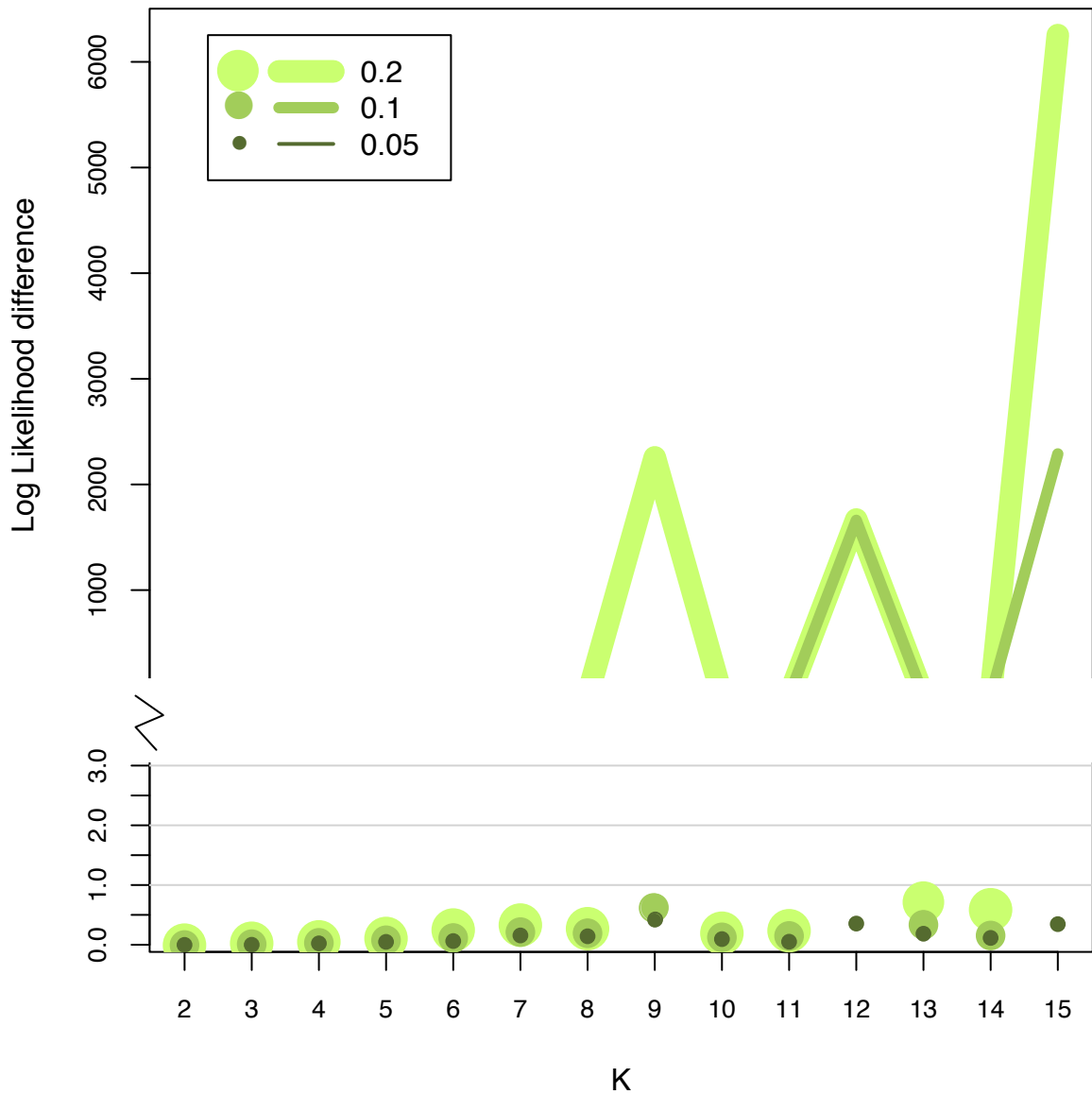

Supplement: S6 Fig — For clarity, the y-axis is shown in two sections. (PDF) [file pgen.1005068.s006.pdf]

Cross Validation errors

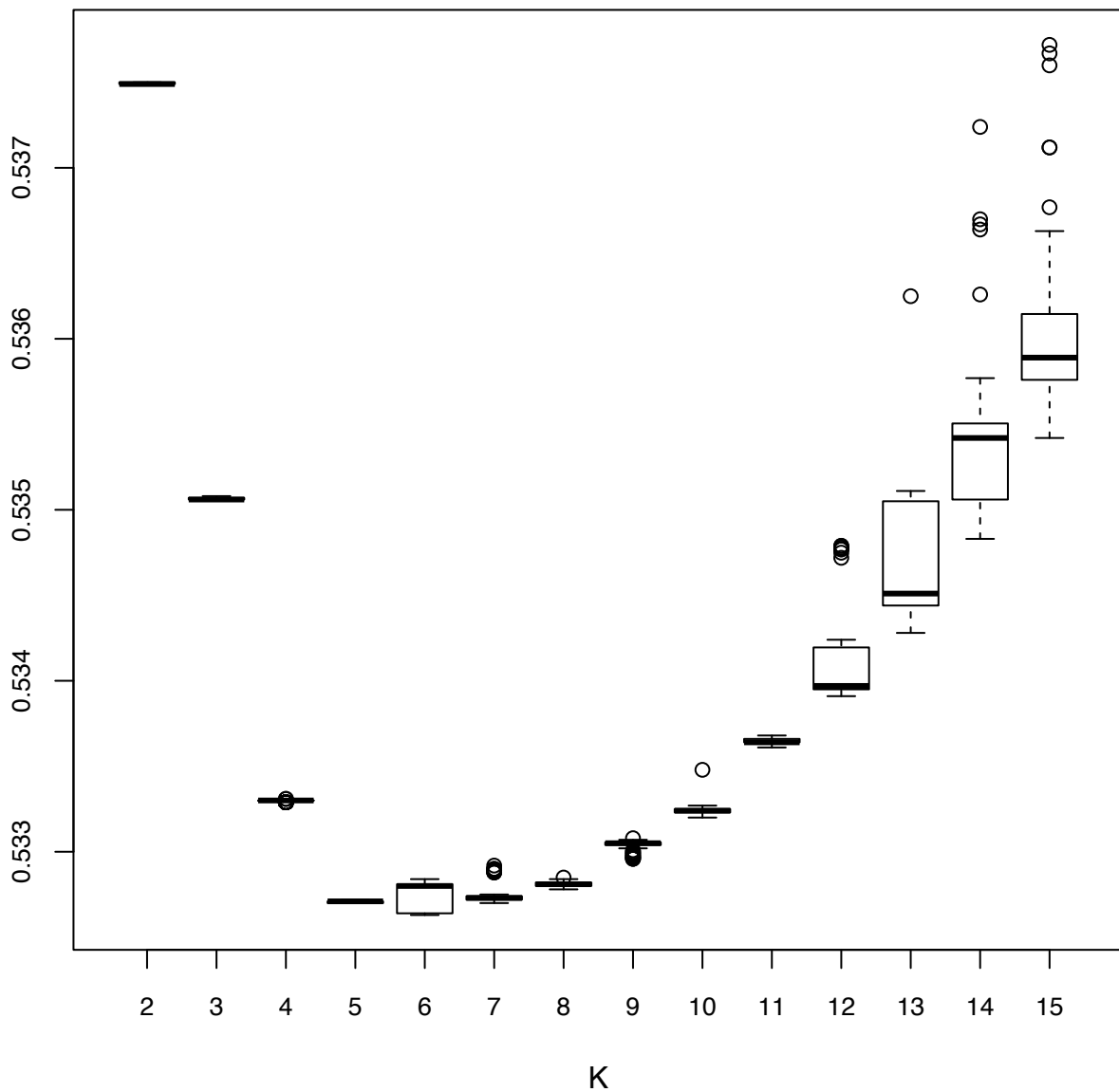

Supplement: S7 Fig — (PDF) [file pgen.1005068.s007.pdf]

A

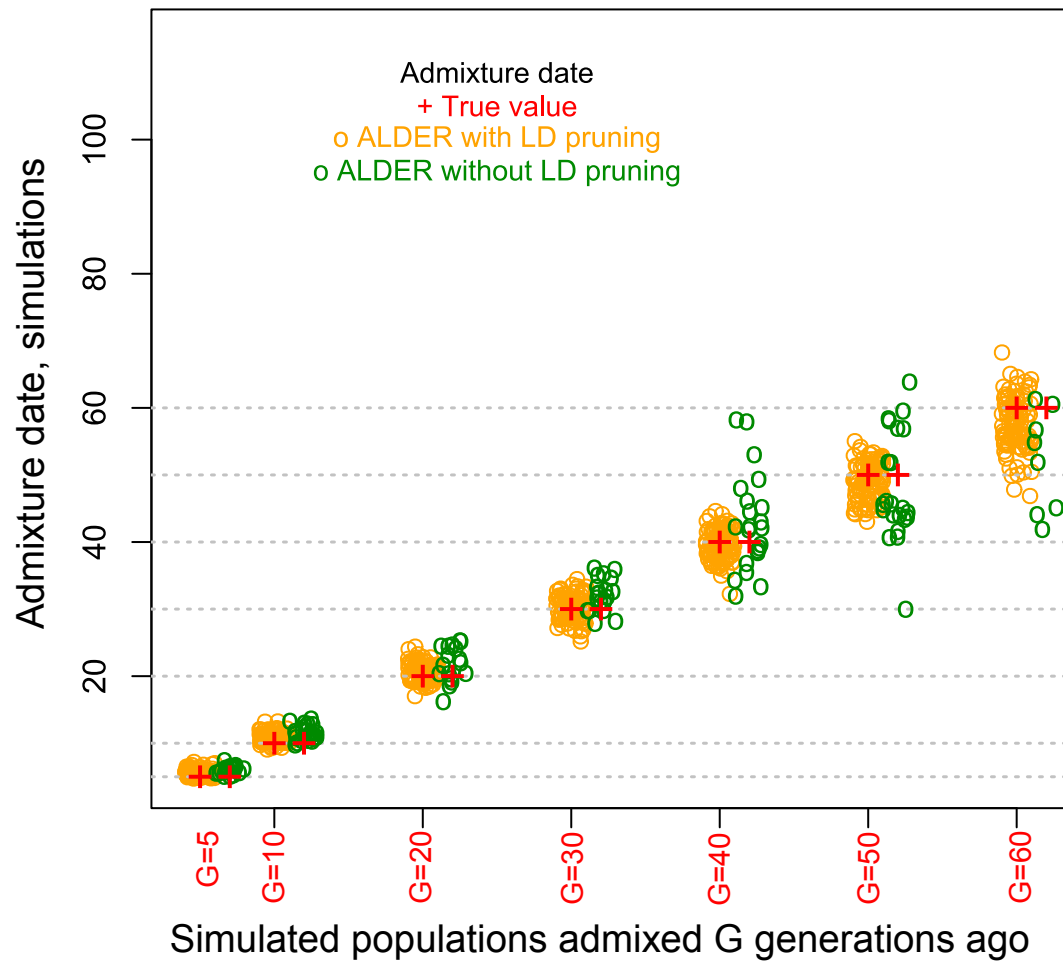

B

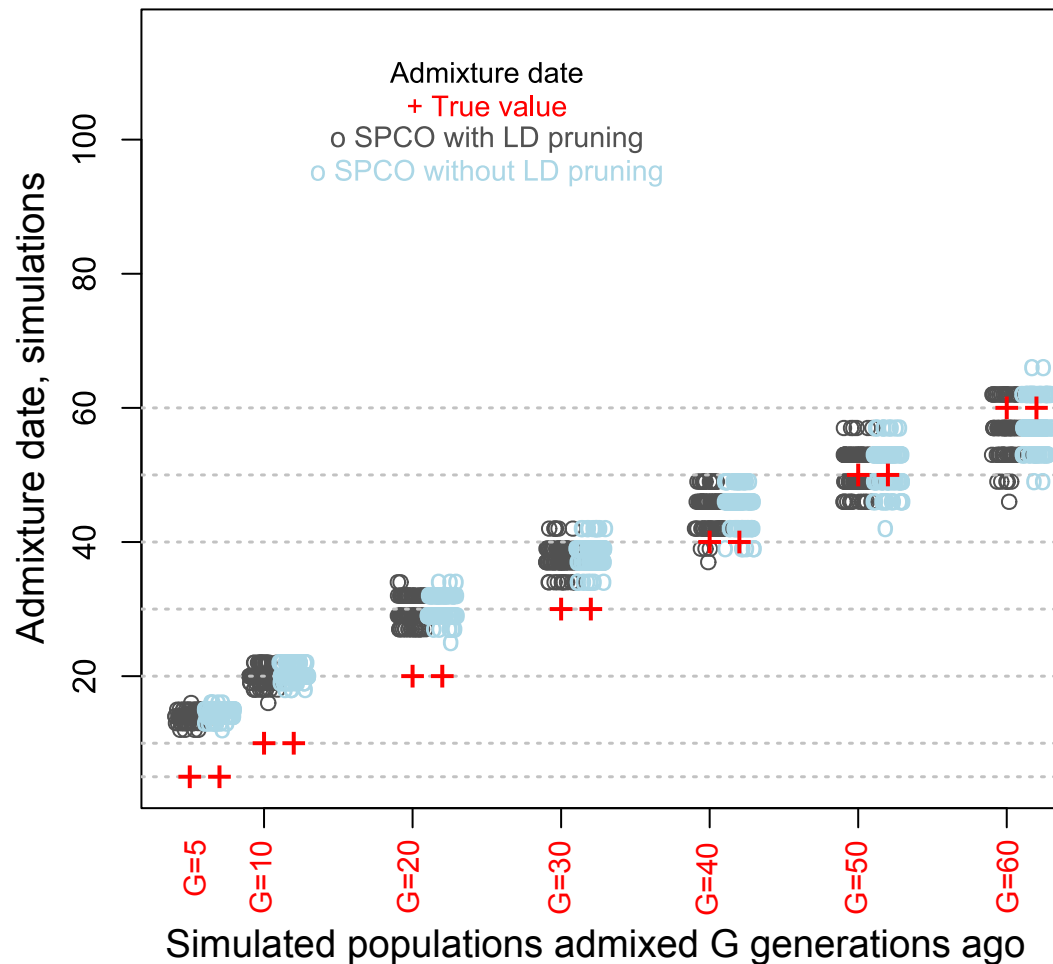

Supplement: S8 Fig — Panel A) ALDER method with and without LD pruning. Panel B) SPCO method with and without LD pruning. (PDF) [file pgen.1005068.s008.pdf]
