# Supplementary material for: The Genetic Legacy of the Expansion of Turkic-Speaking Nomads across Eurasia
Source: PLoS Genet. 2015 Apr 21;11(4):e1005068. doi: 10.1371/journal.pgen.1005068 (PMC4405460; doi:10.1371/journal.pgen.1005068)
Supplement: S2 Fig — Panels A, B, C, and D show sequence of steps in the analysis. (PDF) [file pgen.1005068.s002.pdf]

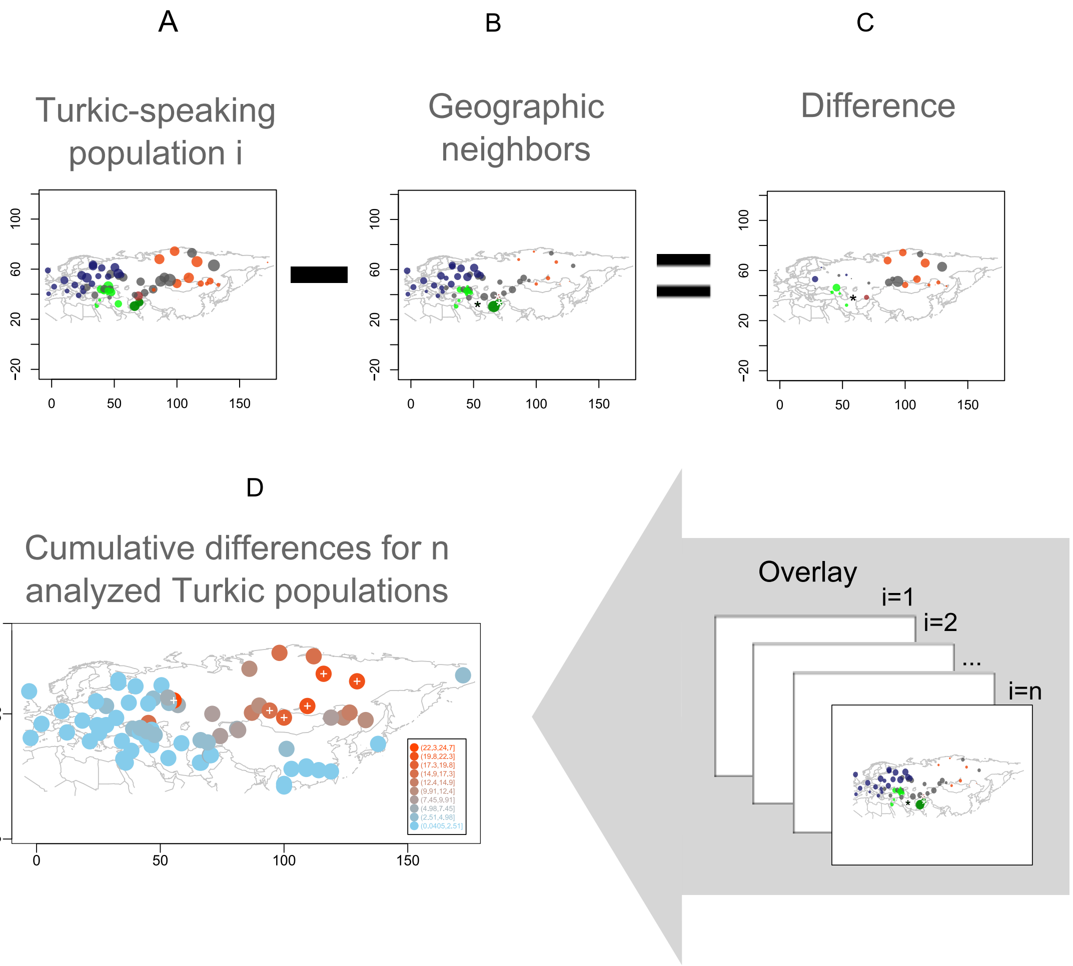
A
B
C
Turkic-speaking population i
Geographic neighbors
Difference
100
60
20
-20
0
50
100
150
D
Cumulative differences for n analyzed Turkic populations
(22.3,24.7]
(19.8,22.3]
(17.3,19.8]
(14.9,17.3]
(12.4,14.9]
(9.91,12.4]
(7.45,9.91]
(4.98,7.45]
(2.51,4.98]
(0.0405,2.51]
Overlay
i=1
i=2
...
i=n
